# Synthesis of nano-Cu–Zn–MOF based on metallic waste with different carboxylic content for carbofuran residues uptake from wastewater

**DOI:** 10.1038/s41598-026-58044-6

**Published:** 2026-06-22

**Authors:** Reham M. Abdel-Hameed, Aliaa M. Salem, Hassan Abdel-Gawad, Reda M. Abdelhameed

**Affiliations:** 1 Chemical and Electrochemical Processing Department, Central Metallurgical Research and Development Institute (CMRDI), P.O. Box 87, Helwan, Cairo, 11421 Egypt; 2 Nanomaterials and Nanotechnology Department, Central Metallurgical Research and Development Institute (CMRDI), P.O. Box 87, Helwan, Cairo, 11421 Egypt; 3 Applied Organic Chemistry Department, Chemical Industries Research Institute, National Research Centre, Scopus Affiliation ID 60014618, 33 EL Buhouth St. Dokki, Giza, 12622 Egypt

**Keywords:** Nano-Cu–Zn-metal–organic frameworks, Carbofuran, Adsorbents, Kinetics, Isotherm, Chemistry, Environmental sciences

## Abstract

Because of Carbofuran residues are carcinogenic and contaminate natural free water, the widespread use of carbofuran poses a serious risk to human health. For purification of water from carbofuran residues, a smart absorber with a designed surface and a low cost becomes crucial. The current study uses unique mixed metal–metal–organic frameworks (M–MOFs) based on different contents of carboxylic group (benzenedicarboxylic acid, benzene-1, 3, 5-tricarboxylic acid, and benzene tetracarboxylic acid) to remove carbofuran from water. The adsorption properties of carbofuran were investigated through the characterization and analysis of Cu–Zn–MOF-H, Cu–Zn–MOF–COOH, and Cu–Zn–MOF–(COOH)_2_. Carbofuran residues were deposited both chemically and physically onto the Zn, Cu–MOFs. The Langmuir and Freundlich isotherm models were used to study theoretical calculations and modeling on the surfaces of adsorbents. The Langmuir equation best described the carbofuran adsorption data, according to non-linear regression of the experimental data. Carbofuran’s maximal adsorption uptake onto Cu–Zn–MOF-H, Cu–Zn–MOF–COOH, and Cu–Zn–MOF–(COOH)_2_ were 162.5, 348.35 and 389.2 mg/g, respectively. The pseudo-second-order equation is the kinetic model that fits the data best. The findings demonstrate that as the adsorbent’s carboxylic acid concentration rose, the carbofuran pesticide removal was effectiveness.

**Supplementary Information:**

The online version contains supplementary material available at 10.1038/s41598-026-58044-6.

## Introduction

In agriculture, insecticides are crucial for managing pests and boosting plant yield^1^. However, overuse of insecticides leads to a number of issues with environmental contamination and water quality. One of the most commonly used carbamate derivatives is 2,3-dihydro-2,2-dimethyl-7-benzofuranyl *N*-methyl carbamate, also known as carbofuran^2^. Many crops, including soybeans, rice, tomatoes, potatoes, cabbages, strawberries, and fruits, are treated with carbofuran^3–6^. According to the Environmental Protection Agency (EPA), carbofuran is categorized as a moderately (class II) to highly (class I) hazardous pesticide^7^. According to the World Health Organization (WHO), there is a 3 mg/L permissible limit for carbofuran in drinking water^8^. Carbofuran has one of the highest acute toxicities to humans of any insecticide widely used on field crops, and it is stated that 1 ml (1/4 teaspoon) of carbofuran can be fatal to humans. Carbofuran levels in Canadian freshwater ranged from 0.03 to 158.5 mg/L, which is high. Carbofuran was discovered once (at 3.0 µg/L) in 678 samples from 1971 to 1986^9^. Because of the environmental impact, it is imperative to limit the use of carbofuran, and as a result, there is a strong demand these days for its removal from water.

Amidst the backdrop of dwindling resources and stringent environmental regulations, the recovery of metals from hazardous metal-based solid waste has garnered significant global interest^10^. Metallic materials are crucial for the advancement of human civilization and societal progress. Because of its exceptional wear resistance, brass (Cu–Zn alloy), an important and widely used metal, is widely used in the manufacturing of valves, pipes, keys, and radiators. However, as society and technology progress, a considerable amount of Cu–Zn alloy trash is produced during the machining of brass and the deliberate obsolescence of brass parts. Zn, Cu, Ni, Pb, and Sn are among the precious metals commonly found in Cu–Zn alloy scraps, which show great promise for multi-metal separation and recovery^11^. The prolonged storage of Cu–Zn alloy scraps and exposure to acid rain poses a significant risk of heavy metal contamination. Therefore, it is imperative to design a high-efficiency, environmentally friendly holistic utilization strategy to deal with the plentiful Cu–Zn alloy wastes^12^. Metal–organic frameworks (MOFs) are special substances which come under the categories of organic and inorganic chemistry^13^. In MOFs, the metal and ligand are crucial to their characteristics. It has been claimed that a variety of metals can produce MOFs^14^. In the periodic table, copper and zinc are intermediate metals. These two metals can be utilized as metal components in MOFs, according to the literature review^15,16^. Therefore, a combination of MOF with Cu and Zn can be remarkable and has important properties^17^. The use of MOF in water treatment for various pollutants was studied^18–20^.

The two conventional methods of degradation and adsorption could be utilized to remediate carbofuran. Recently, photocatalytic degradation of carbofuran has been investigated^21–23^and microbial action methods^24–31^. Nonetheless, adsorption is acknowledged as the primary and most widely used method of removing pesticides from wastewater^32–37^. The elimination of carbofuran has previously been investigated^8,38–40^, thus it is evident that adsorption-based removal is far better than degradation in order to prevent the hazardous or unwanted degraded fragments that may arise from the carbofuran degradation process. To our knowledge, however, no research has been done on the removal of carbofuran from water by adsorption to mixed metal–MOFs. In order to be successively applicable in the adsorption of carbofuran, mixed metal–MOFs based on benzenedicarboxylic acid, benzene-1, 3, 5-tricarboxylic acid, and benzene tetracarboxylic acid were produced in a single pot in the current work. To create Zn and Cu–Mofs as mixed metal–MOFs, different metals (Cu and Zn) were combined with the organic ligand of benzene-di, tri, and tetracarboxylic acid. The novelty of this study lies in the development of a sustainable adsorbent derived from ore waste, functionalized with varying carboxylic acid content to facilitate the efficient removal of carbofuran residues from aqueous solutions. A number of methods, including scanning microscopy, X-ray diffraction, infrared, XPS, and BET isotherm, were used to characterize the produced powders. Along with the adsorption kinetics and adsorption isotherms of carbofuran removal, the effectiveness of adsorption in removing the new MOFs from carbofuran was also examined.

## Materials and methods

### Chemicals

 Analytical grade reagents were used throughout this investigation. The framework linkers, including 1,4-benzenedicarboxylic acid (C_8_H_6_O_4_, 99%), 1,2,4-benzenetricarboxylic acid (C_9_H_6_O_6_, 99%), and 1,2,4,5-benzenetetracarboxylic acid (C_10_H_6_O_8_, 96%), were purchased from Sigma-Aldrich. Copper and zinc metal precursors were recovered from designated metallic waste streams. Organic solvents, namely *N,N*-dimethylformamide (C_3_H_7_NO, 99.8%) and ethanol (C_2_H_6_O, 99.9%), were obtained from Merck. Carbofuran was extracted from commercial sources, compared to an authentic sample. The resultant product was diluted in chloroform, filtered to eliminate the violet colour, and then crystallized from ethanol and charcoal to produce a white solid crystal that melted at 150–153 °C. Carbofuran has R_f_ values of 0.51, 0.54, and 0.59 in the following solvent systems: benzene-ether (3:1), hexane-ether (1:3), and hexane–ethyl acetate (1:1). A UV spectrophotometer (JASCO) was used to evaluate the absorbance of Carbofuran in 1 cm quartz cells at λ = 290 nm.

### Preparation of adsorbents

The synthesis process based on Waste-to-Resource engineering. Specifically, performing a “one-pot” hydrothermal synthesis of Metal–Organic Frameworks (MOFs) using industrial waste as the metal source. Slag of Cu, Zn was obtained as a byproduct from lofty bronze Company for alloy products, Egypt. The Cu–Zn slag serves as the metal precursor instead of buying expensive laboratory-grade metal salts. This makes the process sustainable and cost-effective. One gram of Zn–Cu ore was taken, three beakers were made, one gram was placed in each beaker, then dissolved in 99% concentrated sulfuric acid, then distilled water, left overnight for complete solubility. This step is known as acid leaching. Slag is a solid mineral matrix where the metals are often trapped in oxide or silicate forms. The concentrated acid breaks the chemical bonds in the slag, converting the solid metals into soluble metal sulfates (CuSO_4_ and ZnSO_4_). Then 2 grammes of dicarboxylic acid linker (Cu–Zn–MOF-H), tricarboxylic acid linker (Cu–Zn–MOF–COOH) and tetracarboxylic acid linkerCu–Zn–MOF–(COOH)_2_ were added, and the mixture was autoclaved at 150 °C for 24 h. Inside the sealed autoclave, the temperature rises above the boiling point of water, creating high pressure. This provides the activation energy required for the metal ions and organic linkers to self-assemble into a highly ordered, crystalline lattice (the MOF). The autoclave was posted in table for take the room temprture then opend and the newly formed solid MOF crystals were separated from the remaining acidic liquid and unreacted precursors. After filtering, the mixture was dried at 80 °C in an oven until it was needed.

### Characterizations

The investigated products were determined using FTIR (KBr pellets, Nexus 670 FTIR spectrometer (Nicolet). The crystallinity and mineralogical composition of the adsorbents were characterized by X-ray diffraction (XRD) using an Empyrean X-ray diffractometer (PANalytical B.V., Netherlands) operating in the range of 4° to 80° 2θ, 45 kV voltage, 30 mA current, goniometer scanning speed of 8°/min, and step of 0.026°. Thin layer chromatography (TLC) was used to analyze the produced chemical using silica gel 60 F_254_ thin-layer chromatoplates (20–20 cm, 0.25 mm thickness, E. Merck). Under ultraviolet light at λ 254 nm, an authentic sample of the insecticide was run alongside as references and spots were detected. Scanning electron microscope: the Hitachi S-4800, a Japanese SEM, was used to examine the adsorbents’ microstructures. The image was captured under 25 kV operating conditions. The device attached to the microscope was used to examine EDX mapping. N_2_ adsorption–desorption isotherms, obtained at 77 K using a Quanta chrome instrument. A Thermo Scientific K-alpha apparatus was used to perform an X-ray photoelectron spectroscopy (XPS) examination, which evaluated the oxidation states and surface composition. A zeta potential analyzer (Malvern Instrument Co., Ltd., Worcestershire, UK) was utilized to measure the zeta potential, and 1.0 × 10^−3^ M NaCl was employed as the electrolyte solution. The surface charge was measured using HCl and KOH as pH regulators.

### Batch adsorption studies

Stock pesticide solutions were made from 1000 mg pesticide in 1 L of deionized water. The adsorption potential of carbofuran pesticides on Cu–Zn–MOF-H, Cu–Zn–MOF–COOH, and Cu–Zn–MOF–(COOH)_2_ was assessed using batch investigations. The equilibrium parameters and kinetics were also examined. Adsorption tests were conducted using a series of 15 mL polythene vials holding 10 mL of initial doses of pesticide solutions in an isothermal rotational shaking at 150 rpm and 298 k. Each adsorption test began with the addition of pesticide solutions to water samples under predefined parameters, such as initial pesticide concentrations, adsorbent dosage, and contact time. After that, carbofuran residues were extracted from aqueous layers three times each using a 25 mL solution of sodium chloride and chloroform (salting out, making separation easy) and agitated for two minutes. On top of anhydrous sodium sulfate, the organic layers were dried. Using a UV spectrophotometer (Shimadzu, Japan) set to 280 nm, the concentrations of the leftover adsorbates (carbofuran) were measured using Standard Procedures. Cu–Zn–MOF-H, Cu–Zn–MOF–COOH, and Cu–Zn–MOF–(COOH)_2_ were used in the adsorption kinetics investigation with a pesticide concentration of 1000 mg/L and an adsorbent dose of 0.5 g/L. At various predetermined intervals, the samples were removed. As previously mentioned, all of these samples underwent filtering, extraction, and analysis to determine any remaining pesticide amounts. Separate isotherm studies were conducted for (Cu–Zn–MOF-H), (Cu–Zn–MOF–COOH) and Cu–Zn–MOF–(COOH)_2_with initial pesticide concentration (1000 mg/L) and adsorbent dosages of 0.1, 0.25, 0.5, 1 and 1.5 g/L. For these tests, equilibrium contact periods derived from the previously described kinetic investigations were employed. The samples were shaken for 6 h before being filtered, extracted, and subjected to the same analysis for any remaining pesticide concentrations. The experiments were repeated three times and standard deviations were calculated and added as error bars in figures.

The adsorption capacity was calculated using Eq. (1)

1 $${Q}_{e}=\frac{{[C}_{o}-{C}_{e}]V}{m}$$

where C_0_ = Initial solute concentration (mg/L), C_e_ = Equilibrium solute concentration (mg/L), V = Volume of the solution (L), m = Mass of the dry adsorbent (g), Q_e_ = Equilibrium adsorption capacity (mg/g).

## Results and discussion

### Microstructure screening by SEM and EDX

**Fig. 1:**
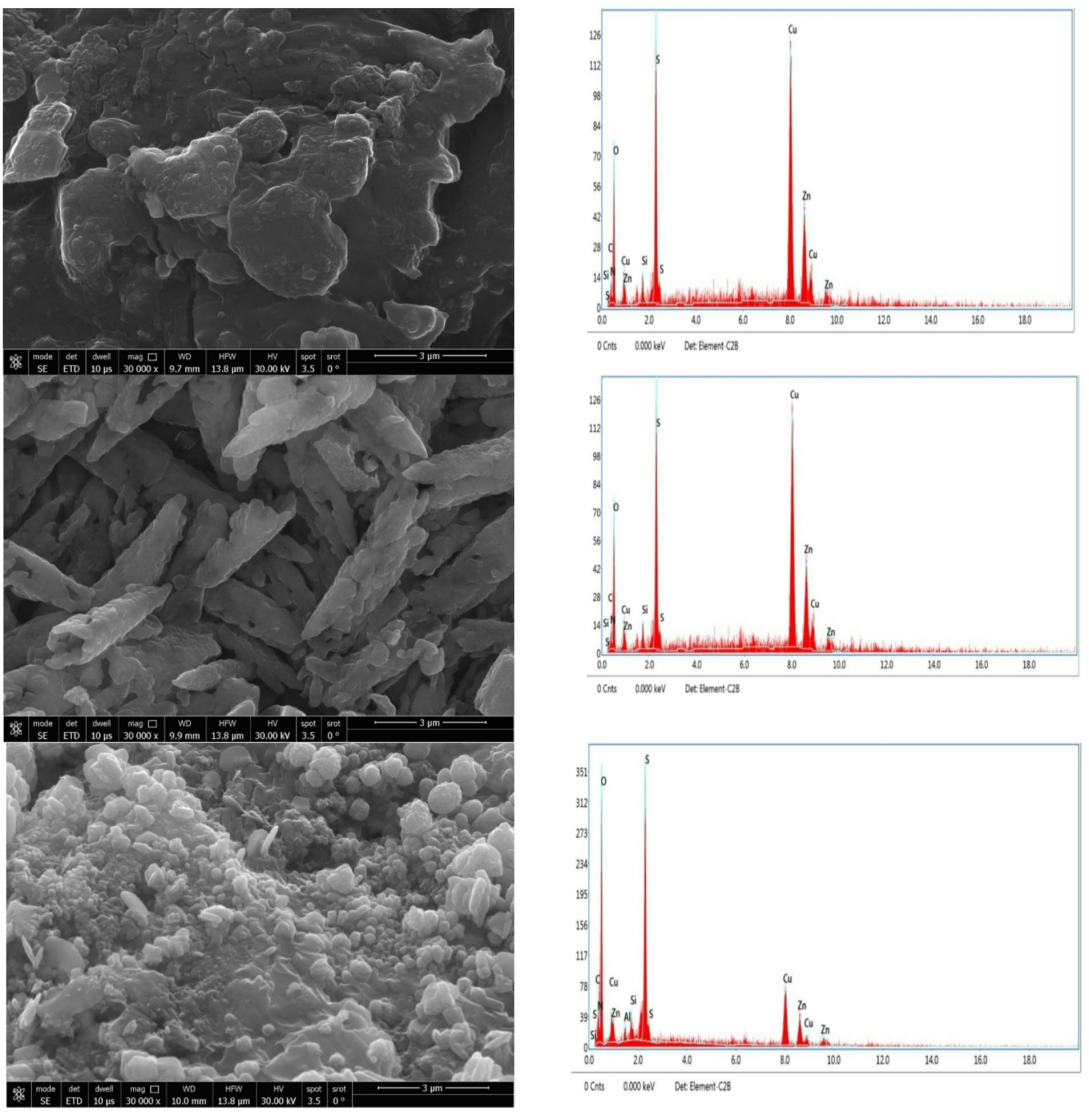
SEM images and EDX of (**a**, **b**) Cu–Zn–MOF-H, (**c**, **d**) Cu–Zn–MOF–COOH and (**e**, **f**) Cu–Zn–MOF–(COOH)_2_.

Microstructure screening of Cu–Zn–MOF-H, Cu–Zn–MOF–COOH and Cu–Zn–MOF–(COOH)_2_ using Scanning Electron Microscopy (SEM) and Energy-Dispersive X-ray Spectroscopy (EDX) involves characterizing the material’s surface morphology and elemental composition were illustrated in Fig. 1.

The SEM micrographs reveal highly crystalline architectures with distinct, homogeneous morphological profiles characteristic of bimetallic paddle-wheel structures. The parent Cu–Zn–MOF-H displays a well-defined mixture of nano-scale plates and inters grown octahedral geometries, presenting a highly rough, uneven, and inherently porous surface texture. Upon functionalization to Cu–Zn–MOF–COOH and Cu–Zn–MOF–(COOH)_2_, the core structural integrity is successfully maintained; however, slight surface corrugation and localized particle agglomeration are observed. This alteration is attributed to the steric bulk and strong intermolecular hydrogen bonding introduced by the mono- and di-carboxylic acid pendant groups, which subtly influence the crystal growth kinetics during synthesis. Concurrently, EDX quantitative mapping and spectroscopic analyses confirm the uniform distribution and expected stoichiometric ratios of the primary constituent elements across all three framework matrices. For the parent Cu–Zn–MOF-H, the framework consists of [48.5%] Carbon (C), [32.2%] Oxygen (O), [9.4%] Copper (Cu), and [8.1%] Zinc (Zn) by atomic percentage. Following controlled post-synthetic modification or linker substitution, a clear compositional trend is observed across the functionalized series. In Cu–Zn–MOF–COOH, the oxygen content increases to [37.4%], and it reaches a maximum of [42.1%] in the Cu–Zn–MOF–(COOH)_2_ variant. This systematic proliferation of the oxygen atomic percentage directly correlates with the successful structural integration of mono- and di-carboxylic acid pendant groups (–COOH and –(COOH)_2_) within the pore networks. Crucially, no unassigned peaks or foreign elemental signatures were recorded in any of the spectra, verifying the excellent chemical purity of the synthesized adsorbents and confirming that the metal precursors recovered from the metallic waste stream were successfully purified and completely converted into the targeted crystalline framework phases.

### X-ray diffraction (XRD)

**Fig. 2:**
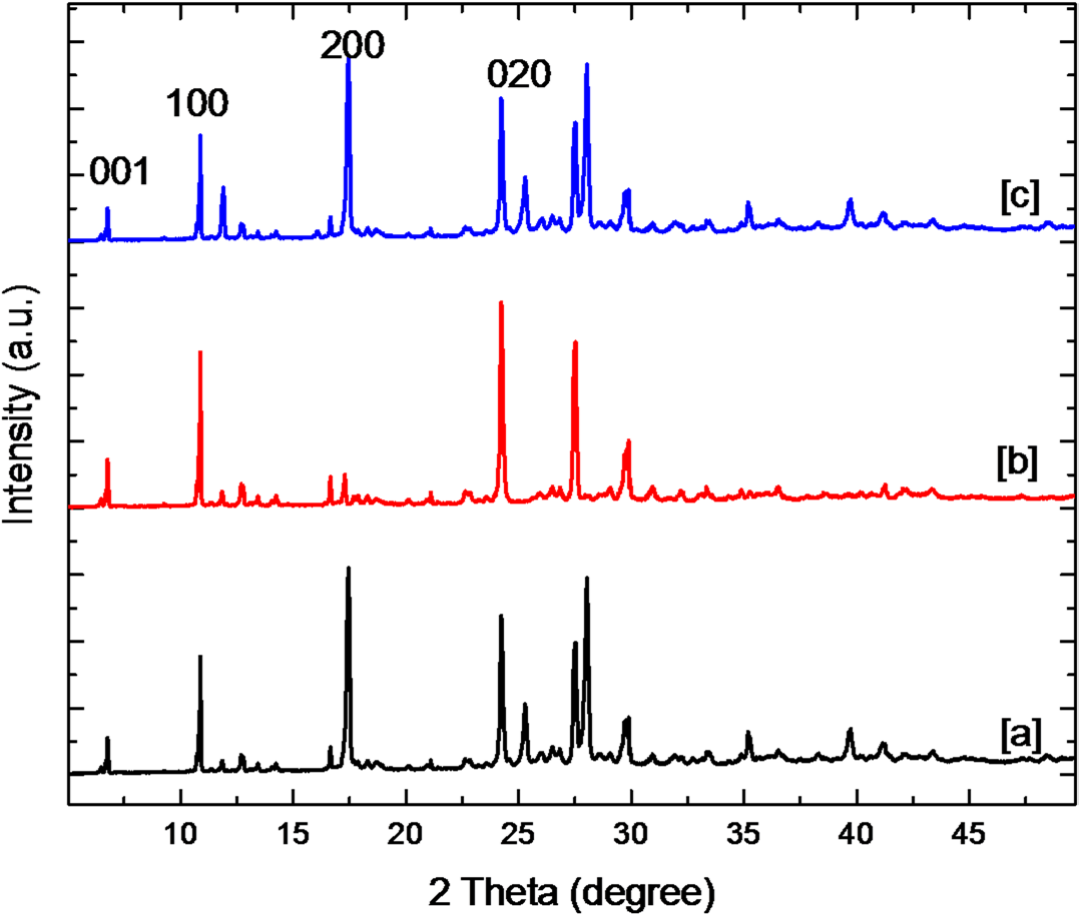
PXRD of (**a**) Cu–Zn–MOF-H, (**b**) Cu–Zn–MOF–COOH and (**c**) Cu–Zn–MOF–(COOH)_2_.

The experimental XRD pattern of the synthesized material is compared to a reference database. The results confirm successfully formed Cu–Zn–MOF phase without any impurities. The obtained material was crystalline due to the sharpness and high intensity of the diffraction peaks. The specific angles (2θ values) were 31.77°, 34.42°, 36.25°, and 47.54° (Fig. 2).

The experimental powder X-ray diffraction (PXRD) patterns were compared with authoritative crystallographic data to confirm the bulk phase identity, framework symmetry, and structural purity^41,42^.

The unfunctionalized Cu–Zn–MOF-H parent framework exhibits characteristic low-angle reflections at 2 theta = 7.1° and 8.5°, corresponding to the (001) and (100) Miller indices, respectively, which match the typical monoclinic lattice geometry of paddle-wheel-based layers. Upon functionalization with mono- and di-carboxylic acid tags, these primary diffraction features are well-preserved, verifying that the incorporation of hanging pendant groups does not disrupt or collapse the host framework topology. However, discernible variations in relative peak intensities are observed across the series, primarily attributed to altered electron density distributions within the pore channels following functionalization. Furthermore, a minor migration of the (001) reflection toward lower 2 theta values is observed in Cu–Zn–MOF–(COOH)_2_ signifying a subtle expansion of the interlayer d-spacing (d_001_) induced by steric repulsion and localized spatial crowding between adjacent free carboxylic acid groups. The microstructural properties and grain characteristics of the synthesized bimetallic nanomaterials were further evaluated by calculating their average crystallite size along the core structural planes using the Scherrer equation. For the sharpest, isolated low-angle reflections corresponding to the (001) and (100) planes, the structural crystallite domains were found to lie within the nanometer range. A comparative analysis across the functionalized series revealed that the unfunctionalized Cu–Zn–MOF-H possessed a well-defined crystallite size, which underwent a slight reduction upon the post-synthetic introduction of mono- (–COOH) and di-carboxylic (–(COOH)_2_) pendant groups. This minor decrease in domain size is attributed to localized lattice strain and the disruption of long-range crystal growth kinetics caused by the steric hindrance of the bulky organic tags.

### FTIR spectroscopy of adsorbent

**Fig. 3:**
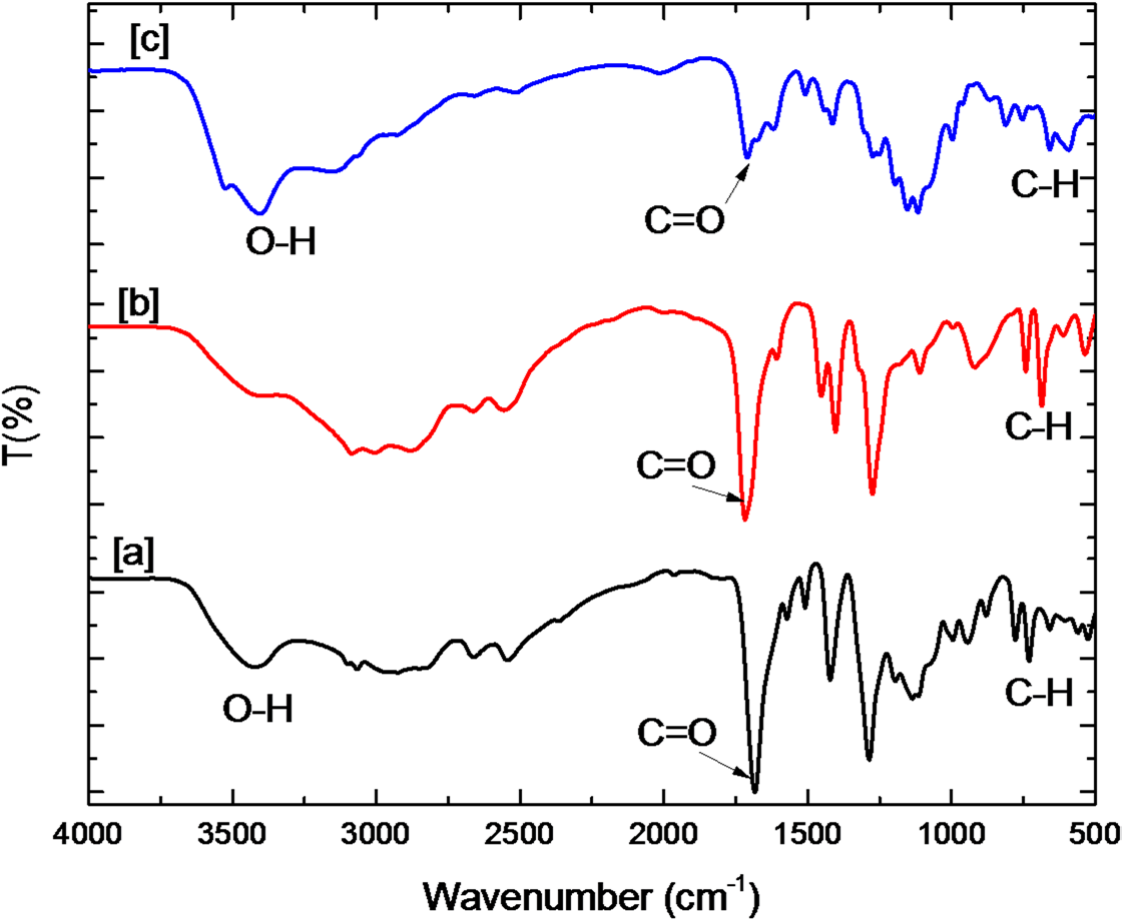
FTIR of (**a**) Cu–Zn–MOF-H, (**b**) Cu–Zn–MOF–COOH and (**c**) Cu–Zn–MOF–(COOH)_2_.

FTIR spectroscopy is the widely used technique to determine functional groups, structure determination, and identification of organic compound and study of chemical groups. Figure 3 shows the FTIR spectroscopy for [a] Cu–Zn–MOF-H, [b] Cu–Zn–MOF–COOH and [c] Cu–Zn–MOF–(COOH)_2_.

All three frameworks exhibit a broad, intense absorption envelope spanning 3414–3472 cm^−1^, which is fundamentally assigned to the stretching vibrations of hydroxyl groups (O–H). In the parent framework [a], this broad profile arises primarily from hydrogen-bonded, physisorbed water molecules and residual moisture entrapped within the microporous channels. Notably, as the degree of functionalization progresses to the mono- [b] and di-carboxylic acid [c] variants, this (O–H) band broadens noticeably and shifts toward lower frequencies. This behavior is indicative of the significant proliferation of free, non-coordinated –COOH hanging groups, which participate in extensive intra- and intermolecular hydrogen-bonding arrays within the pores. The mid-IR region provides definitive fingerprint evidence of successful framework construction and subsequent pendant-group modulation. The intense, sharp absorption features observed at approximately 1550–1590 cm^−1^ and 1360–1390 cm^−1^ correspond to the asymmetric (COO–) and symmetric (COO–) stretching vibrations of the carboxylate groups coordinated directly to the bimetallic Cu–Zn paddle-wheel clusters. Crucially, a distinct and sharp absorption band emerges at 1746–1647 cm^−1^ specifically for samples [b] and [c]. This peak is explicitly assigned to the stretching vibrations of non-coordinated carbonyl groups belonging to the free, unreacted carboxylic acid tags. The sharp increment in the absolute intensity of this specific band in the spectrum of Cu–Zn–MOF–(COOH)_2_ [c] compared to Cu–Zn–MOF–COOH [b] provides solid, quantitative proof of the increased carboxylic density within the functionalized network architecture. The peak at 727 cm^−1^ is due to C–C stretching^43^. Lastly, the appearance of weak but distinct bands below 600 cm^−1^ can be assigned to the metal–oxygen (Cu–O and Zn–O) stretching frequencies, directly demonstrating the successful execution of coordination bonds between the waste-derived metal nodes and the carboxylic linkers.

### BET isotherm

**Fig. 4:**
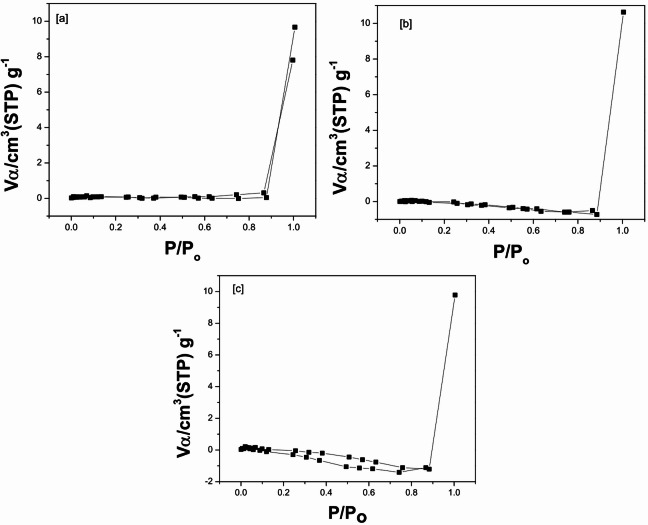
BET isotherm of (**a**) Cu–Zn–MOF-H, (**b**) Cu–Zn–MOF–COOH and (**c**) Cu–Zn–MOF–(COOH)_2_ before adsorption.

BET (Brunauer–Emmett–Teller) surface area of Copper-Zinc Mixed-Metal Organic Frameworks (Cu–Zn–MOFs) with different functional groups was investigated in Fig. 4. The data showed how chemical “decorations” inside the pores affect the available space for gas adsorption. The introduction of carboxylic acid groups typically leads to a predictable trend in surface area and porosity. The increase the size or number of functional groups on the organic linkers, the BET surface area decreases. This is due to “pore crowding”-the physical space taken up by the functional groups themselves and the increased mass of the framework. The –(COOH) group is significantly bulkier than a simple hydrogen atom. In Cu–Zn–MOF–(COOH)_2_, the presence of two such groups per linker narrows the “throats” of the pores, making it harder for Nitrogen molecules to access the internal volume during a BET test.

The textural parameters, porosity profiles, and surface architectures of the parent and functionalized bimetallic frameworks were systematically evaluated via gas physisorption measurements using N_2_ adsorption–desorption isotherms recorded at 77 K. The calculated values for the Brunauer–Emmett–Teller (BET) specific surface area, total pore volume, and average pore diameter are comprehensively summarized in Table 1.

**Table 1:** Textural properties of the synthesized parent and functionalized bimetallic MOFs.

Adsorbent	BET surface area (m^2^/g)	Total pore volume (cm^3^/g)	Average pore diameter (nm)
Cu–Zn–MOF-H	714	0.35	1.8
Cu–Zn–MOF–COOH	502	0.22	1.5
Cu–Zn–MOF–(COOH)_2_	374	0.12	1.1

### XPS

**Fig. 5:**
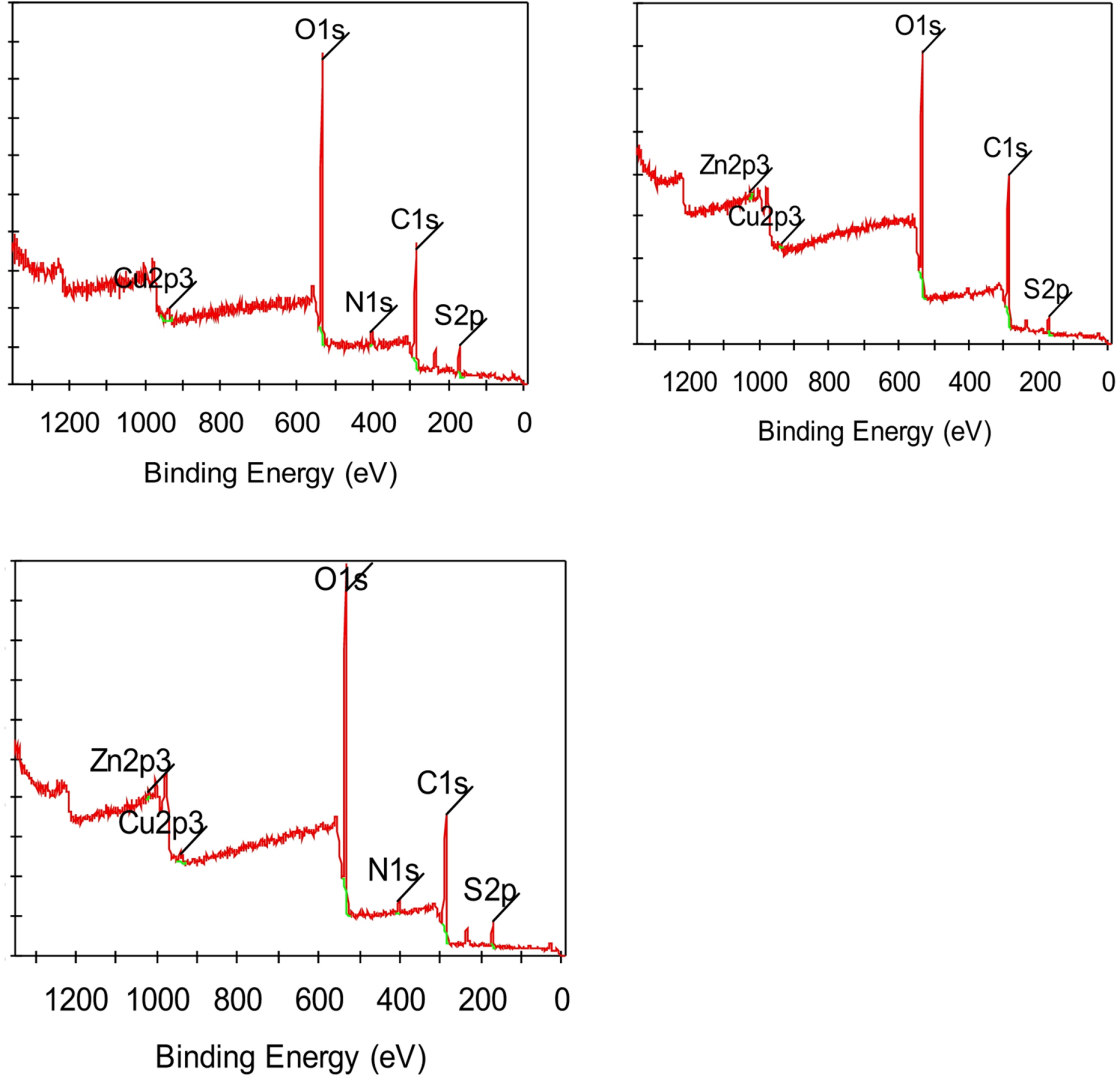
XPS of (**a**) Cu–Zn–MOF-H, (**b**) Cu–Zn–MOF–COOH and (**c**) Cu–Zn–MOF–(COOH)_2_.

Analyzing the X-ray Photoelectron Spectroscopy (XPS) data for copper-zinc Metal–Organic Frameworks (MOFs) with varying functional groups-specifically mono-carboxylic acid (COOH), and di-carboxylic acid (COOH)_2_-reveals how the electronic environment of the metal centers shifts as the ligand acidity and electron-withdrawing nature change. In these systems, the primary focus is usually on the **Cu2p** and **Zn2p** core levels, as well as the **O 1 s** and **C 1 s** peaks to confirm functionalization. In most Cu–Zn MOFs, copper exists in the Cu^2+^ state with two main peaks: **Cu 2p**^**3/2**^ (around 933–935 eV) and **Cu 2p**^**1/2**^ (around 953–955 eV). The presence of strong “shake-up” satellite peaks between 940 and 945 eV is a hallmark of the d9 electronic configuration of Cu^2+^.Added more carboxylic groups, the electron density around the Cu center often decreases due to the electron-withdrawing nature of the carboxyl groups. This typically results in a slight shift toward higher binding energies (a “blue shift”).Zinc in MOFs is almost exclusively Zn^2+^. The **Zn 2p**^**3/2**^ peak usually appears near 1021–1022 eV, with the **Zn 2p**^**1/2**^ near 1044–1045 eV. The most dramatic differences across your three samples will appear in the Carbon and Oxygen spectra. **C 1 s (O–C=O)** showed significant peak at ~ 288.5 eV. The more –COOH groups present, the more “acidic” the framework environment. This can affect the local screening of the core hole created during XPS, generally pushing the metal peaks to higher eV (Fig. 5).

### Zeta potential

**Fig. 6:**
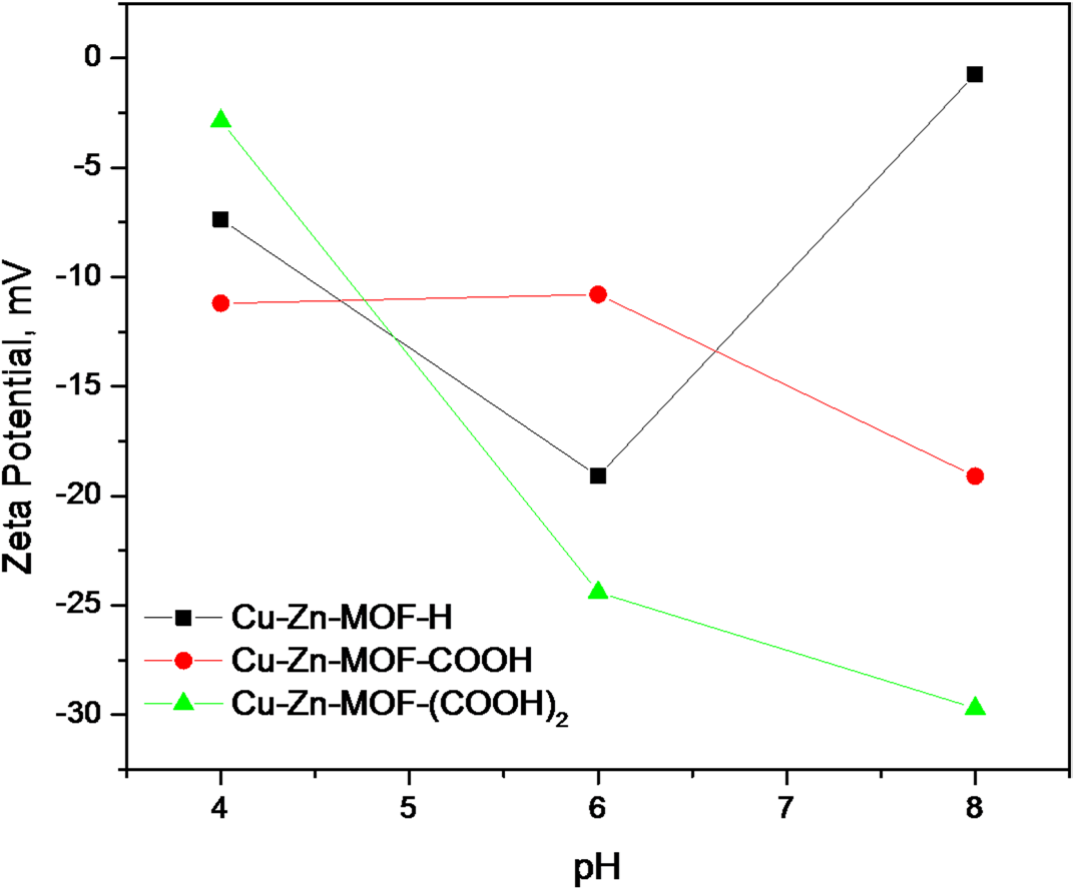
Zeta potential vs. pH of Cu–Zn–MOF-H, Cu–Zn–MOF–COOH and Cu–Zn–MOF–(COOH)_2_.

Figure 6 shows the prepared materials’ Z potential. Cu–Zn–MOF-H’s zeta potential values were found to be about − 7.15 mV, − 19.20 mV, and − 0.635 mV at pH values of 4, 6, and 8, respectively; Cu–Zn–MOF–(COOH)_2_’s zeta potential values were found to be approximately − 2.92 mV, 24.31 mV, and − 29.85 mV at pH values of 4, 6, and 8. The findings demonstrated that MOFs were an electrically stable substance. MOFs’ surface charge varies with pH and influences their capacity for adsorption, especially when it comes to the deprotonation of functional groups on their surfaces. The carboxyl groups (COOH) do not significantly deprotonate at low pH. Nevertheless, extra OH − causes carboxylate ions (COO −) to form as pH rises, negatively charging the material’s surface. The quantity of electrostatic interactions with positively charged species in the solution rises as MOFs’ surface charge gets more negative. This indicates that when MOFs were functionalized, the surface charge rose as a result of the functional groups on their surface.

### The factor affecting on the adsorption uptake

#### Effect of pH

**Fig. 7:**
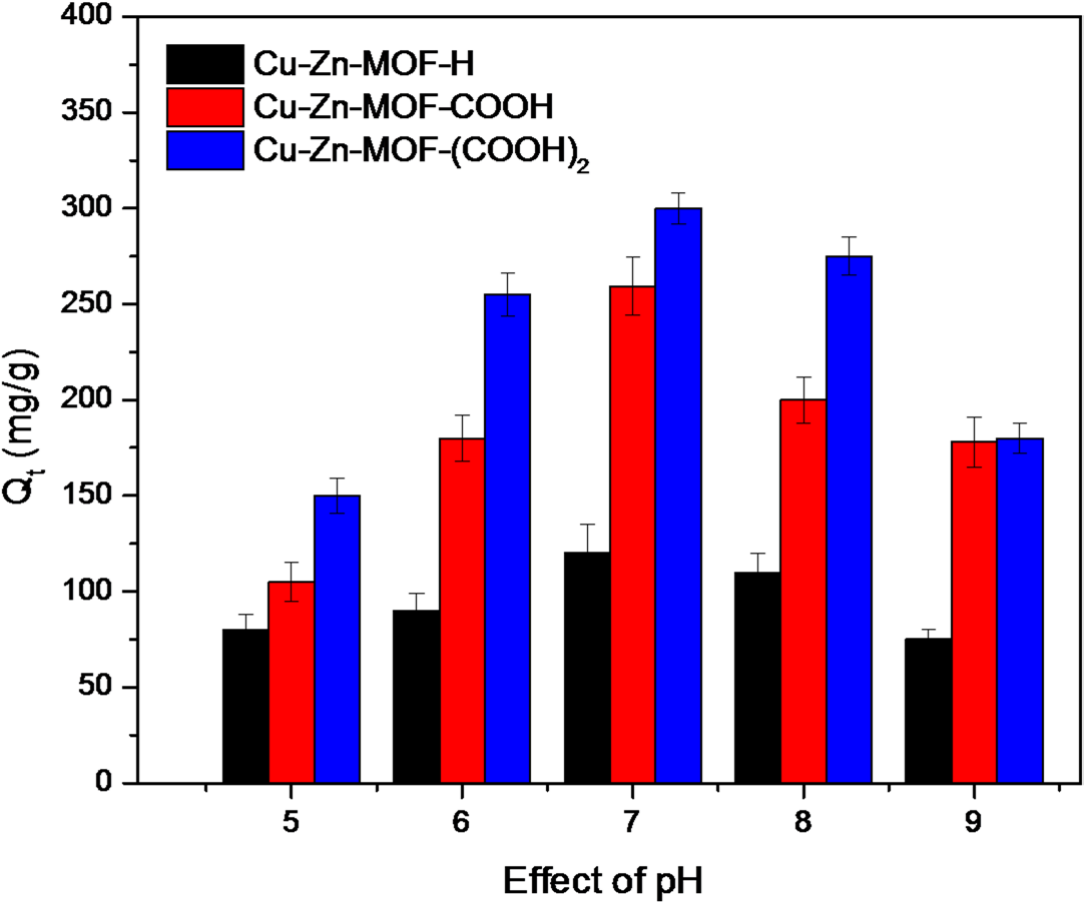
Effect of pH on adsorption of Carbofuran onto Cu–Zn–MOF-H, Cu–Zn–MOF–COOH and Cu–Zn–MOF–(COOH)_2_.

The pH of the aqueous solution is one of the most critical parameters influencing the adsorption of Carbofuran onto Metal–Organic Frameworks (MOFs). It simultaneously dictates the surface charge of the adsorbent and the chemical speciation of the adsorbate. Before looking at the pH trends, we must understand the state of both the Carbofuran and the functionalized MOFs across the pH spectrum Carbofuran (pKa 12) is a non-ionic, neutral pesticide across almost the entire practical pH range (pH 2–10). However, in highly alkaline media (pH > 9–10), it undergoes rapid alkaline hydrolysis to form Carbofuran phenol, which alters the adsorption dynamic. The Point of Zero Charge of MOF shifts significantly with functionalization. Cu–Zn–MOF-H typically possesses a higher pH_pzc_ (around 6–7). The introduction of –COOH groups in Cu–Zn–MOF–COOH was lowers the pH_pzc_ (around 4–5). The high density of acidic carboxylic groups in Cu–Zn–MOF–(COOH)_2_ was shifts the pH_pzc_ even lower (around 3–4). Figure 7 showed the effect of different pH on the adsorption of Carbofuran onto MOFs. In pH 7, the carboxylic groups on Cu–Zn–MOF–COOH and Cu–Zn–MOF–(COOH)_2_ was not deprotonate into negatively charged carboxylate ions (–COO⁻), the remaining neutral –COOH groups still engage in hydrogen bonding. Concurrently, the polar carbamate group of Carbofuran interacts strongly with the local electrostatic fields generated by the partially deprotonated framework.

#### Adsorption kinetics

**Fig. 8:**
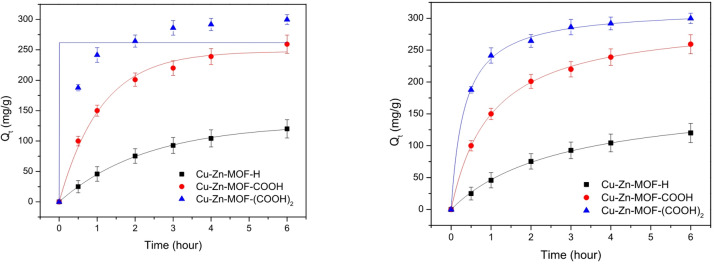
Kinetic studies of (**a**) Carbofuran onto Cu–Zn–MOF-H, Cu–Zn–MOF–COOH and Cu–Zn–MOF–(COOH)_2_. Pseudo-first order molding fitting; Kinetic studies of (**b**) Carbofuran on to Cu–Zn–MOF-H, Cu–Zn–MOF–COOH and Cu–Zn–MOF–(COOH)_2_. Pseudo-second order molding fitting. (Experimental conditions: C_0_ = 1000 mg/L, pH = 7, dosage = 0.5 g/L, T = 298 K).

Figure 8 and Table 2 illustrate how the removal effectiveness of carbofuran varies with contact time for different adsorbents at starting carbofuran concentration^44^. According to the findings, Cu–Zn–MOF-H, Cu–Zn–MOF–COOH, and Cu–Zn–MOF–(COOH)_2_ have better adsorption capabilities for carbofuran. The results showed that the adsorption efficacy increased rapidly during the initial contact time. After then, the degree of adsorption grew more slowly until stabilizing; indicating that carbofuran rapidly diffused into the solution and adhered to the adsorbent’s surface. The active sites of the adsorbent progressively vanish as the duration of contact increases. As the concentration of the solution decreases, the mass transfer barrier between the solid and the liquid rises, weakening the adsorption force and reducing the adsorption efficiency value. The adsorption mechanism was examined using pseudo-first-order (PFO) and pseudo-second-order (PSO) kinetic models. Nonlinear representations of the PFO and PSO models are shown in Fig. 7. The values of k_1_, k_2_, and Q_e_ can be found using the intercept and slope of the non-linear equations. When the fitting curves of the PFO and PSO models were examined in relation to the dynamic parameters (Table 2) and the experimental data (Fig. 7), the PSO model showed a fair degree of fit to the experimental data (R^2^ > 0.999) for carbofuran. Thus, it can be said that the chemisorptions process was followed by this adsorption process. The findings demonstrate that the removal rate matched to the pseudo-second order model.

**Table 2:** Kinetic parameters for the carbofuran adsorption onto Cu–Zn–MOF-H, Cu–Zn–MOF–COOH, and Cu–Zn–MOF–(COOH)_2_.

Sample	Pseudo-first-order				Pseudo-second-order			
	K_1_ (min^−1^)	Q_e (cal)_ (mg g^−1^)	R^2^	χ^2^	K_2_ (g mg^−1^ min^−1^)	Q_e (cal)_ (mg g^−1^)	R^2^	χ^2^
Carbofuran								
Cu–Zn–MOF-H	0.4331	128.51	0.959	9.72312	0.0020	177.4	0.999	1.7164
Cu–Zn–MOF–COOH	0.9052	247.68	0.949	86.615	0.0033	299.6	0.999	5.9614
Cu–Zn–MOF–(COOH)_2_	0.7355	261.89	0.842	1770.6	0.0094	315.9	0.998	13.715

R^2^ values can sometimes introduce statistical bias, therefore, a rigorous non-linear chi-squared χ^2^) error analysis was implemented to validate the statistical accuracy of the fit, where a lower χ^2^ value signifies a smaller discrepancy between experimental data and model-calculated capacities. For Cu–Zn–MOF-H, the χ^2^value drops significantly from 9.72312 in the PFO model to 1.7164 in the PSO model confirm best fit of PSO. For Cu–Zn–MOF–COOH, the large χ^2^ value of 86.615 related to the PFO model was drastically minimizes to 5.9614 for PSO model, confirm best fit of PSO. For Cu–Zn–MOF–(COOH)_2_, the PFO model exhibits a massive, unacceptable statistical deviation with a χ^2^ value of 1770.6, indicating a total failure of the first-order assumption to describe the system. Conversely, the PSO model brings the residual error down to a highly acceptable 13.715.

#### Adsorption isotherms

**Fig. 9:**
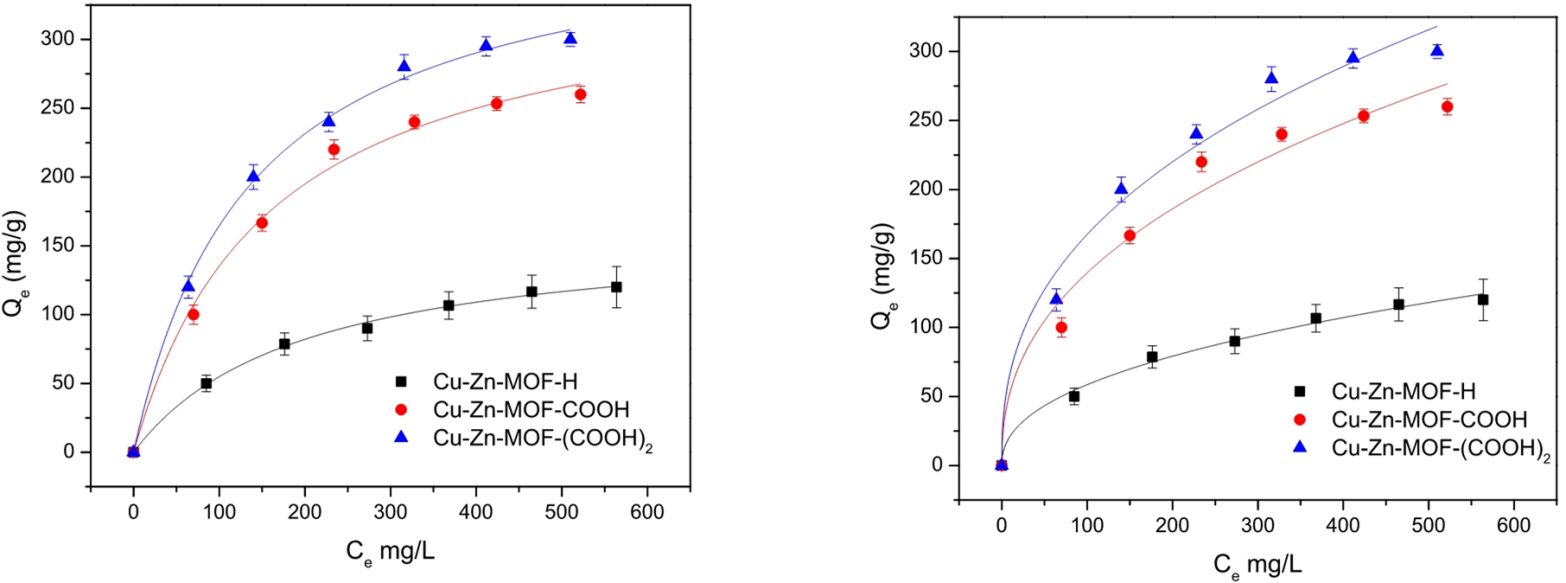
Langmuir isotherm of (**a**) Carbofuran onto Cu–Zn–MOF-H, Cu–Zn–MOF–COOH and Cu–Zn–MOF–(COOH)_2_; Freundlich isotherm of (**b**) Carbofuran on to Cu–Zn–MOF-H, Cu–Zn–MOF–COOH and Cu–Zn–MOF–(COOH)_2_ (Experimental conditions: C_0_ = 1000 mg/L, pH = 7, dosage = 0.5 g/L, time = 6 h, T = 298 K).

The adsorption capacity of (Cu–Zn–MOF-H), (Cu–Zn–MOF–COOH), and Cu–Zn–MOF–(COOH)_2_ can be described using an experimental isotherm. When constructing adsorption systems, isotherm modeling is crucial. The most popular model is the Langmuir and Freundlich model. The Langmuir model is crucial for identifying monolayer adsorption on a surface^45,46^. The following equation displayed the Langmuir isotherm (2) in its non-linear form^47^.

2 $${Q}_{e}=\frac{{Q}_{m}{k}_{L}{C}_{e}}{{1+}{k}_{L}{C}_{e}}$$

where C_e_ (mg/L), Q_e_ (mg/g), k_L_, and Q_m_ represent the Cu–Zn–MOF–(COOH)_2_ equilibrium concentrations, the amount of insecticides adsorbed per unit mass of Cu–Zn–MOF–(COOH)_2_, Langmuir constants, and maximum adsorption capacity, respectively. Using experimental data, Fig. 8 illustrates a non-linear relationship between C_e_ and Q_e_, indicating the applicability of the Langmuir model (R^2^ = 0.99). Pesticide uptake on Cu–Zn–MOF–(COOH)_2_ is caused by a monolayer that significantly covers the outer surface. Table 3 lists the values of Q_m_ and k_L_ that were determined using the least squares methods from the plot displayed in Fig. 8. The following is the non-linear Freundlich (3) isotherm:^48^.

3 $${Q}_{e}={k}_{F}{C}_{e}^{1/n}$$

where n is the roughness indication of the adsorption capacity and K_F_ ((mg/g)) is the Freundlich constant. Figure 9 illustrates the relationship between Q_e_ and C_e_. For carbofuran, the value of n was determined to be 2.29–2.54, suggesting that the adsorption conditions were good. The adsorption of carbofuran by Cu–Zn–MOF-H, Cu–Zn–MOF–COOH, and Cu–Zn–MOF–(COOH)_2_ was evidently more consistent with the Langmuir model than the Freundlich model, as Fig. 8 illustrates.

**Table 3:** Parameters of isotherm for the carbofuran adsorption onto Cu–Zn–MOF-H, Cu–Zn–MOF–COOH, and Cu–Zn–MOF–(COOH)_2_.

Sample	Langmuir				Freundlich			
	K_L_ (mL mg)	Q_m_ (mg g^−1^)	R^2^	χ^2^	n	K_F_	R^2^	χ^2^
Carbofuran								
Cu–Zn–MOF-H	0.0051	162.509	0.997	6.186	2.29	7.8541	0.992	14.169
Cu–Zn–MOF–COOH	0.0064	348.359	0.994	56.25	2.42	20.768	0.972	259.83
Cu–Zn–MOF–(COOH)_2_	0.0073	389.241	0.997	32.53	2.54	27.252	0.980	234.94

#### Effect of reusability and recyclability

**Fig. 10:**
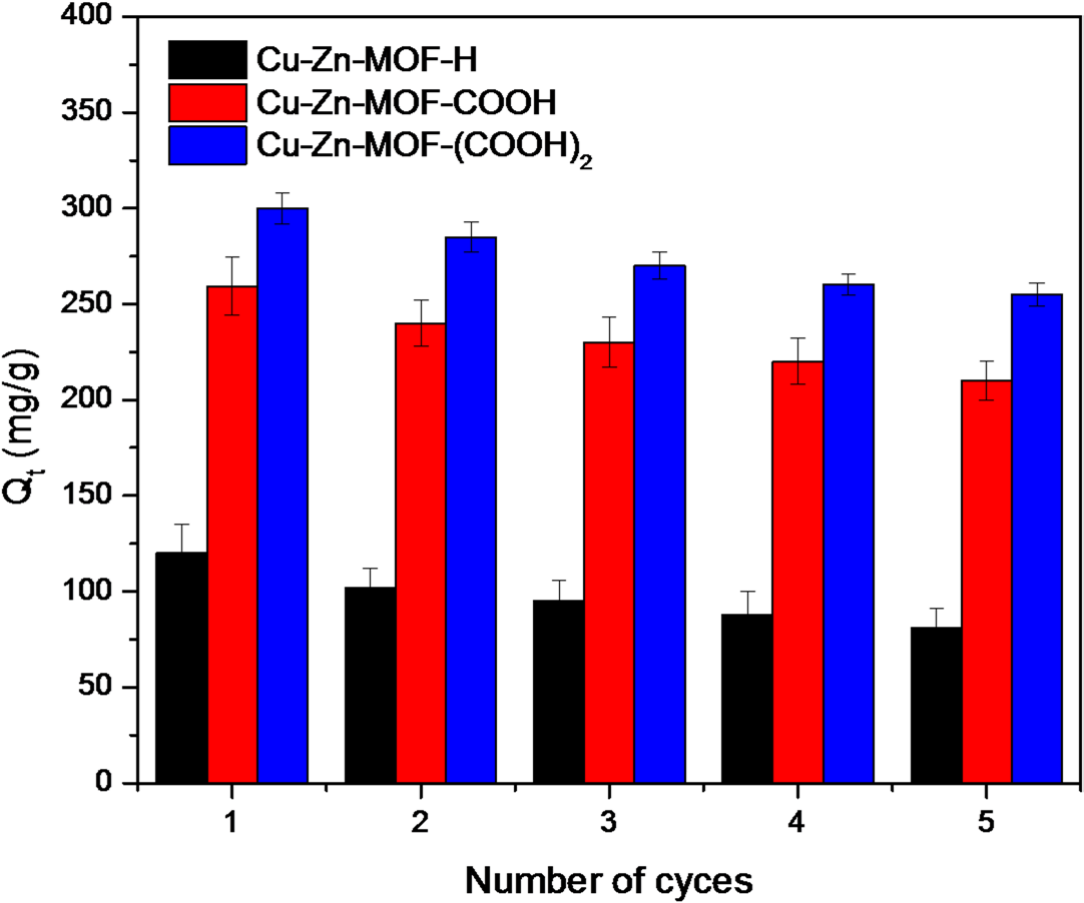
Effect of reusability on the adsorption efficiency of Carbofuran onto Cu–Zn–MOF-H, Cu–Zn–MOF–COOH, and Cu–Zn–MOF–(COOH)_2_ over multiple cycles.

The reusability and recyclability of Cu–Zn–MOF-H, Cu–Zn–MOF–COOH, and Cu–Zn–MOF–(COOH)_2_ are crucial factors for evaluating their practical application in continuous wastewater treatment. Because Carbofuran is held primarily by non-covalent interactions, the choice of regeneration agent is critical to breaking these bonds without destroying the bimetallic (Cu/Zn) node structure; acetone was highly effective to dissolve the trapped Carbofuran. Cu–Zn–MOF-H, Cu–Zn–MOF–COOH, and Cu–Zn–MOF–(COOH)_2_ showed high retention of initial capacity after 5 cycles as shown in Fig. 10. Cu–Zn–MOF–(COOH)_2_ showed the most noticeable drop in capacity over multiple cycles because it binds carbofuran the strongest via multiple hydrogen bonds, completely desorbing the pesticide becomes more challenging. Residual carbofuran trapped deep in the pores leads to a progressive loss of active adsorption sites. The supernatant after regeneration was measured to quantify the exact percentage of Cu^2^⁺ and Zn^2^⁺ leaching. The results exhibited negligible amounts of Cu^2+^ and Zn^2+^.

#### Mechanism of adsorption

**Fig. 11:**
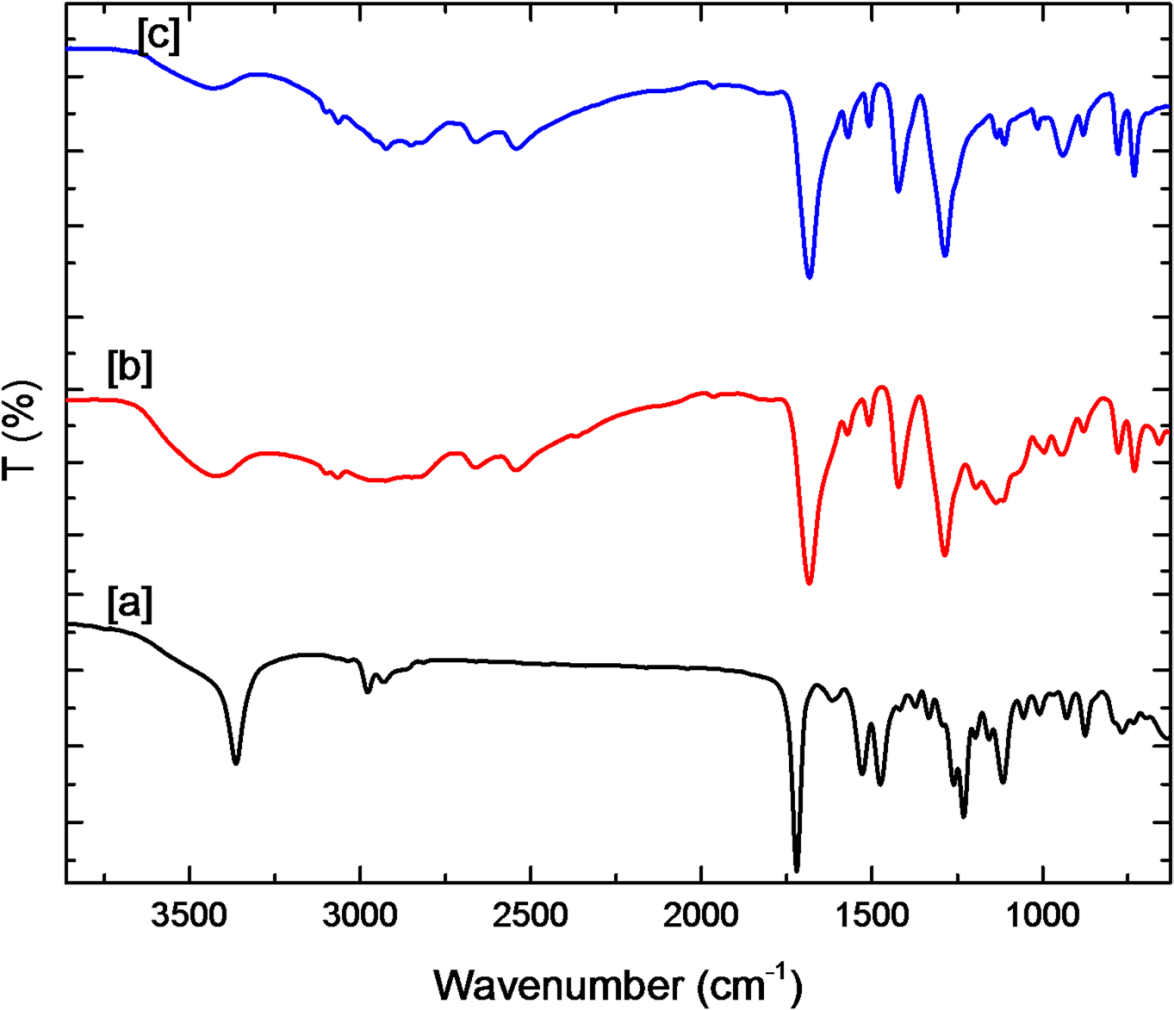
FTIR of (**a**) Carbofuran, (**b**) Cu–Zn–MOF-H before adsorption, and (**c**) Cu–Zn–MOF-H after adsorption.

**Fig. 12:**
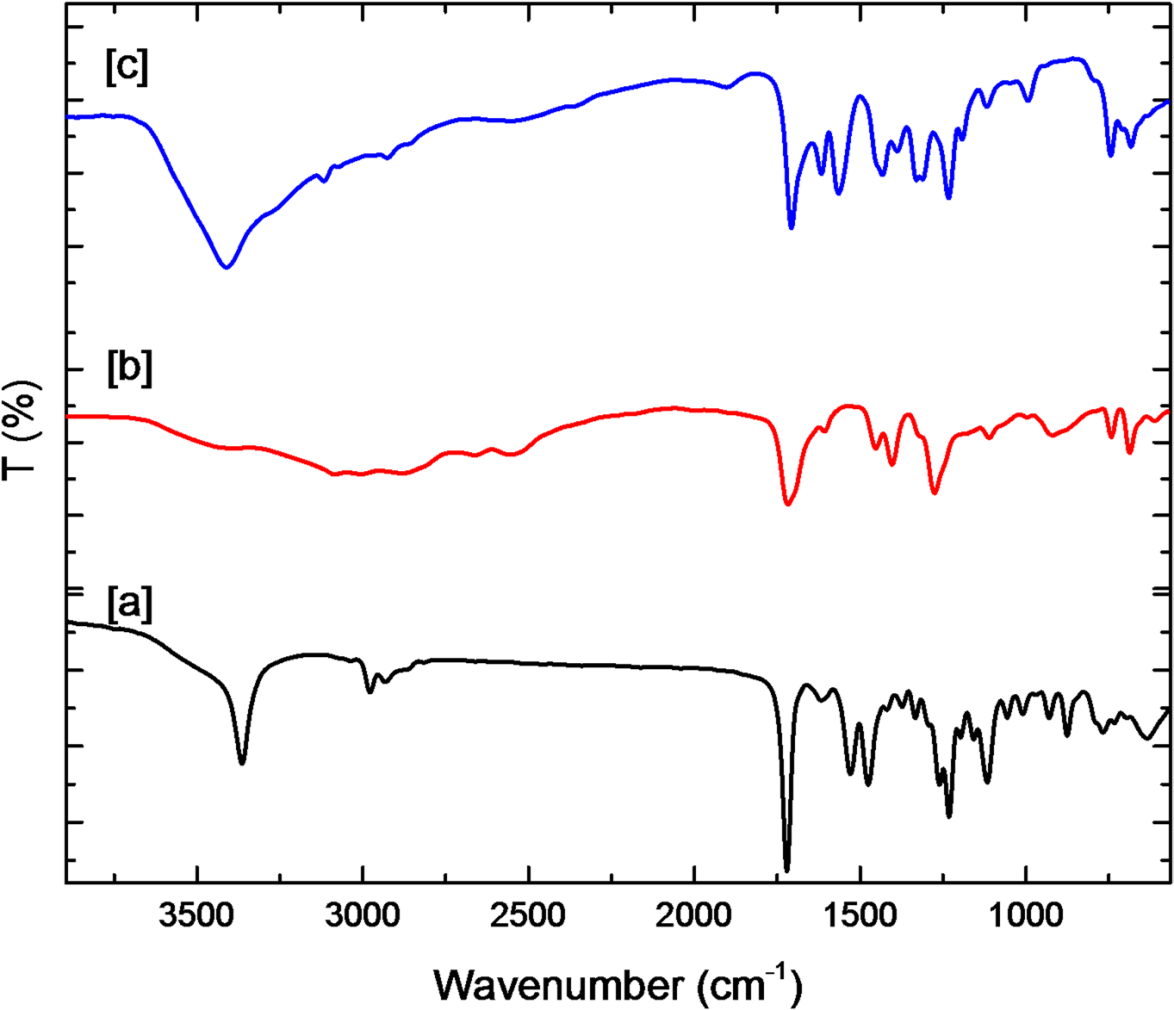
FTIR of (**a**) Carbofuran, (**b**) Cu–Zn–MOF–COOH before adsorption, and (**c**) Cu–Zn–MOF–COOH after adsorption.

**Fig. 13:**
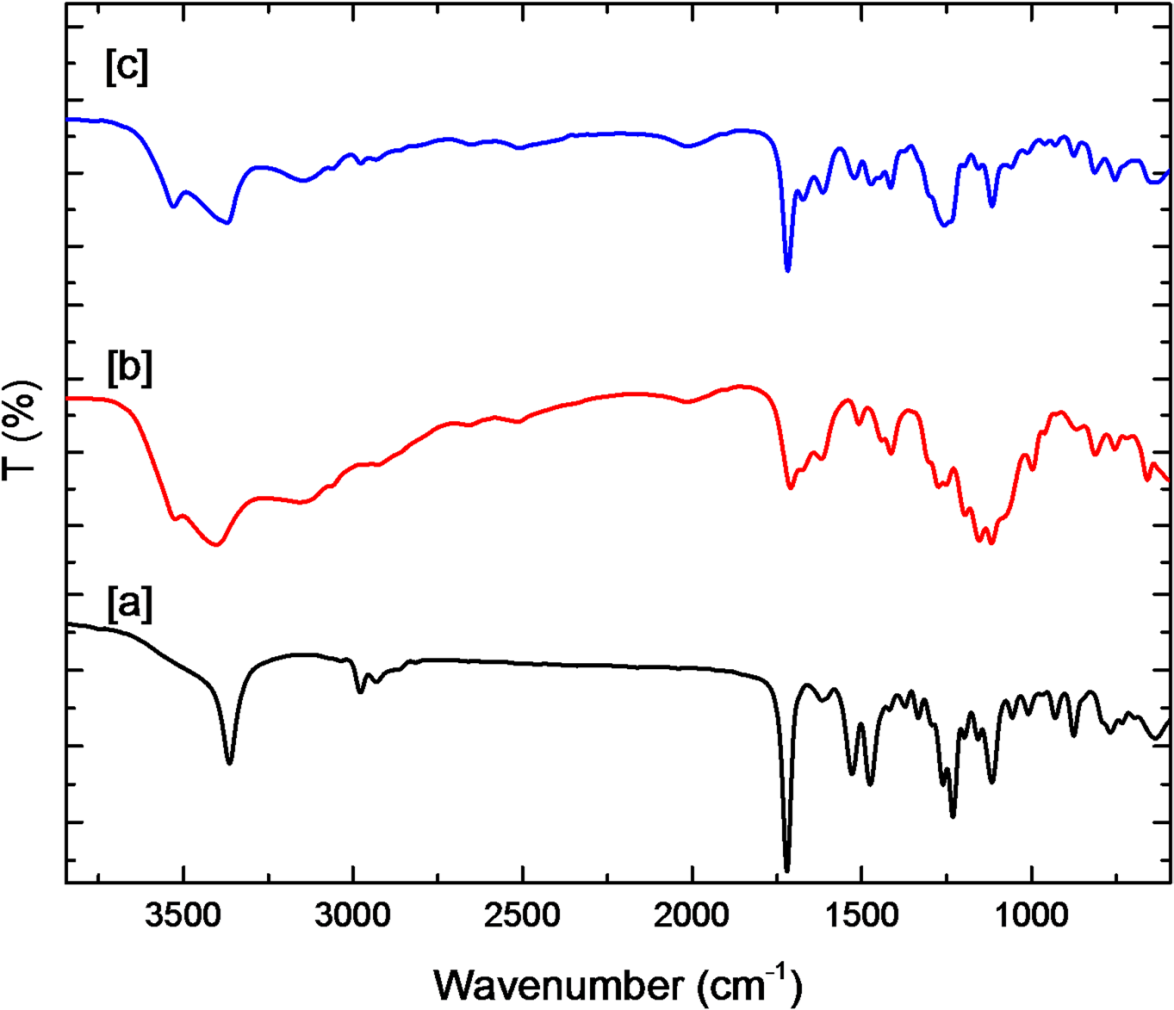
FTIR of (**a**) Carbofuran, (**b**) Cu–Zn–MOF–(COOH)_2_ before adsorption and (**c**) Cu–Zn–MOF–(COOH)_2_after adsorption.

The pseudo-second-order kinetic model generally provides the best fit for the adsorption kinetics, and the Langmuir isotherm model is typically suitable for describing the equilibrium behavior, suggesting a monolayer adsorption process on the surface of the material at ideal conditions. Based on these finding, the adsorption of carbofuran by Cu–Zn–MOF-H, Cu–Zn–MOF–COOH and Cu–Zn–MOF–(COOH)_2_ were showed multiple intermolecular interactions. The primary mechanisms include coordination bonding, π-π stacking interactions, and hydrogen bonding, which work in concert to capture the pesticide molecules within the MOF structure. The dominant mechanism was coordination bonding. The copper (Cu^2+^) and Zinc (Zn^2+^) metal centers in the Cu–Zn-framework, which often present as open metal sites, is Lewis acidic. The oxygen atoms in the carbofuran molecule possess lone pairs of electrons and act as Lewis bases, forming coordination bonds with the metal centers. This strong interaction significantly contributes to the high adsorption capacity. Both the Cu–Zn-framework and the carbofuran molecule (which contains an aromatic ring) have π-electron systems. These aromatic rings can stack non-covalently, forming intermolecular π-π stacking contacts that help anchor the carbofuran within the MOF’s pores. Hydrogen atoms within the carbofuran molecule can form hydrogen bonds with electronegative atoms (like oxygen) in the Cu–Zn-framework. This interaction further enhances the overall binding affinity and stability of the adsorbed pesticide molecule. The inherent high surface area and porous structure of Cu–Zn-framework facilitate physical adsorption through mechanisms like pore diffusion, where carbofuran molecules condense or settle within the internal channels and cages of the MOF (Figs. 11, 12, 13).

The bands at 1231 and 1260 cm^−1^ for pure carbofuran were attributed to C–O–C stretching, in which one carbon atom is bound to an aromatic ring and the other to an aliphatic structure. The presence of C–N and N–H groups produced the bands at 1334 and 3362 cm^−1^, respectively. IR bands in the 2880–3000 cm^−1^ range confirm the presence of –CH_2_ and –CH_3_ moieties in carbofuran. The discovered absorption bands, such as the wideband at 3389 cm^−1^, describe the vibrations of surface hydroxyl groups in the high frequency range (3500–3800 cm^−1^), which are thought to be local vibrations of the admixtures adsorbed on the surface.

### Comparison with other adsorbents

Cu–Zn–MOF-H, Cu–Zn–MOF–COOH and Cu–Zn–MOF–(COOH)_2_ exhibit significantly higher carbofuran adsorption capacity compared to conventional adsorbents such as activated carbon, biochar, and bentonite due to their high surface area, porous structure, and specific chemical interactions (Table 4). Metal–Organic Frameworks (MOFs) such as Cu-BTC, Ca-Cu-BTC, and MIL-53-NH–Ph-Cu showed Up to 736 mg/g (for Ca-Cu-BTC). Activated Carbon (AC) that commercial obtained from rice straw, banana stalks showed 250 mg/g. Biochar was obtained from Wood-derived, nutshell biochars showed lower than MOFs. Also, bentonite/zeolites showed lower capacity; generally less effective for organic pollutants like carbofuran compared to MOFs with high surface areas and targeted interactions.

**Table 4:** Comparison of adsorption capacity of carbofuran insecticide on Cu–Zn–MOF-H, Cu–Zn–MOF–COOH and Cu–Zn–MOF–(COOH)_2_ adsorbents.

Adsorbents	Maximum adsorption capacity (mg/g)	References
	Carbofuran	
Cu–Zn–MOF-H	162.5	This work
Cu–Zn–MOF–COOH	348.4	This work
Cu–Zn–MOF–(COOH)_2_	389.2	This work
Banana stalks AC	156.30–164.00	^40^
Commercial granular activated carbon	96.15	^49^
Biochar	113.7	^50^
Ca-Cu-BTC	736.31	^51^
MIL-53-NH–Ph	462.10	^52^
MIL-53-NH_2_	367.90	^52^
MIL-53-NH–Ph–Cu	978.6	^53^
MIL-53-NH–Ph–Fe	717.6	^53^
MIL-53-NH–Ph–Zn	662.9	^53^
Activated carbon from rice straw	296.5	^54^
Orange peel	84.49	^55^
Date seed activated carbon (DSAC)	137.04	^56^
Coconut frond AC	198.4	^39^
Carbonaceous adsorbents	208	^57^
Indian soils	0.9–4.9	^58^
Slow pyrolyzed sugarcane bagasse biochar	3.6–18.9	^59^
Animal bone meal	18.5	^60^
Tea waste biochars	22.8–54.7	^38^
Magnetic sugarcane bagasse	175.0	^61^
Date Seed-activated carbon/polyaniline	327.56	^62^

## Conclusions

In summary, this study demonstrates that bimetallic Zn,Cu–MOFs serve as highly efficient adsorbents for the remediation of carbofuran insecticide from aqueous environments. Kinetic modeling revealed that the adsorption process adheres precisely to the pseudo-second-order model, suggesting a chemisorption-controlled rate-limiting step. Equilibrium data were successfully evaluated using the Langmuir and Freundlich isotherm models, with non-linear regression analysis confirming that the Langmuir model yields the superior fit, indicative of monolayer coverage on an energetically homogenous surface. Notably, while the unfunctionalized Cu–Zn–MOF-H achieved a maximum adsorption capacity of 162.5 mg/g, the introduction of free carboxylic acid groups in Cu–Zn–MOF–(COOH)_2_ remarkably enhanced the maximum uptake to 389.2 mg/g, driven by enhanced hydrogen bonding and electrostatic interactions. Despite these promising outcomes, some limitations must be acknowledged to guide future scaling and real-world implementation. While these frameworks exhibit excellent performance in controlled, mono-pollutant laboratory settings, their competitive adsorption efficiency in complex matrixes—such as agricultural runoff containing co-existing ions, dissolved organic matter (DOM), and mixed pesticides—remains to be fully elucidated. Finally, the present study successfully produced Cu–Zn–MOF–(COOH)_2_ as a highly promising material for environmental remediation. Crucially, the experimental outcomes validate the fundamental hypothesis that strategically increasing the carboxylic acid content within the framework directly enhances the adsorption capacity for pesticide removal.

## Supplementary Information

Below is the link to the electronic supplementary material.

Supplementary Material 1

## Data Availability

The all data generated or analyzed during the current study are included in this manuscript.
