# Supplementary material for: Synthesis of nano-Cu–Zn–MOF based on metallic waste with different carboxylic content for carbofuran residues uptake from wastewater
Source: Sci Rep. 2026 Jun 22;16:19354. doi: 10.1038/s41598-026-58044-6 (PMC13287582; doi:10.1038/s41598-026-58044-6)
Supplement: Supplementary file 1 — Supplementary Material 1 [file 41598_2026_58044_MOESM1_ESM.docx]

**[a] Cu-Zn-MOF-H**

**[b] Cu-Zn-MOF-COOH**

**Cu-Zn-MOF-(COOH)_2_**

**Figure 1S:** XPS of **[a] Cu-Zn-MOF-H, [b] Cu-Zn-MOF-COOH and [c] Cu-Zn-MOF-(COOH)_2_**

**Calibration Curve and Linearity**

A stock solution of carbofuran was prepared and sequentially diluted to yield a series of working standard solutions across a concentration range of [100-1000] mg/L. The maximum absorption wavelength was λ=290 nm

- The absorbance was plotted against concentration to construct the calibration curve.
- The linear regression equation was determined to be:
- The method exhibited excellent linearity within the investigated working range, yielding a correlation coefficient (R^2^) of **0.95**, which complies with the accepted threshold for quantitative spectrophotometric analysis.

**Limits of Detection (LOD) and Quantification (LOQ)**

The Limit of Detection (LOD) and Limit of Quantification (LOQ) were calculated statistically based on the standard deviation of the response and the slope of the calibration. The calculated **LOD** was determined to be **156 mg/L**. The calculated **LOQ** was determined to be **474 mg/L**.

**Figure 2S: Calibration Curve [a] and Linearity [b]**
